# Cross-Sectional Study on the Association between Dietary Patterns and Sarcopenia in Elderly Patients with Chronic Kidney Disease Receiving Conservative Treatment

**DOI:** 10.3390/nu15234994

**Published:** 2023-12-01

**Authors:** Hiroyuki Inoshita, Daisuke Asaoka, Kei Matsuno, Naotake Yanagisawa, Yusuke Suzuki, Katsumi Miyauchi

**Affiliations:** 1 Department of Nephrology, Juntendo Tokyo Koto Geriatric Medical Center, Tokyo 136-0075, Japan; 2 Department of Gastroenterology, Juntendo Tokyo Koto Geriatric Medical Center, Tokyo 136-0075, Japan; 3 Department of Respiratory Medicine, Juntendo Tokyo Koto Geriatric Medical Center, Tokyo 136-0075, Japan; 4 Medical Technology Innovation Center, Juntendo University, Tokyo 113-8421, Japan; 5 Department of Nephrology, Juntendo University Faculty of Medicine, Tokyo 113-8421, Japan; yusuke@juntendo.ac.jp; 6 Department of Cardiology, Juntendo Tokyo Koto Geriatric Medical Center, Tokyo 136-0075, Japan

**Keywords:** vegetable-based diet, sarcopenia, CKD, variety score dietary

## Abstract

Sarcopenia is a poor prognostic factor in patients with chronic kidney disease (CKD). Adequate dietary patterns are important for preventing sarcopenia; however, evidence regarding the underlying association between sarcopenia and diet is insufficient. Therefore, in this study, we aimed to investigate the association between sarcopenia and dietary patterns in CKD patients receiving conservative treatment. In this cross-sectional study, 441 patients with conservative CKD were examined using the Asian Working Group for Sarcopenia diagnostic criteria. CKD was defined as an eGFR of <60 mL/min/1.73 m^2^ present for >3 months. The participants were divided into sarcopenia and non-sarcopenia groups, and dietary patterns were compared between the two groups using the dietary variety score, a simple dietary survey method that investigates the weekly frequency of consumption of 10 food groups. Logistic regression analysis for CKD G3 showed that female sex (odds ratio (OR): 0.166, 95% confidence interval (CI): 0.086–0.320), increased body mass index (OR: 0.663, 95% CI: 0.590–0.745), and almost daily consumption of green/yellow vegetables (OR: 0.350, 95% CI: 0.176–0.695) were positively associated with non-sarcopenia. Although further prospective studies are required, the results suggest that low frequent consumption of vegetables is associated with sarcopenia in patients with CKD.

## 1. Introduction

Sarcopenia is the loss of skeletal muscle mass and physical function that occurs with advancing age. This issue is especially significant in aging societies, such as Japan, as it profoundly affects the quality of life of the elderly, leading to increased susceptibility to immobility, falls, and hospitalizations and ultimately to higher mortality rates [1].

The term “sarcopenia” was coined by Rosenberg in 1989, and the European Working Group on Sarcopenia in Older People (EWGSOP) subsequently proposed diagnostic criteria for sarcopenia using walking speed, grip strength, and muscle mass as indicators [2]. However, due to skeletal differences between Europeans and Asians, there was a need for sarcopenia diagnostic criteria specific to Asians. Therefore, the Asian Working Group for Sarcopenia (AWGS) was established in 2013 and proposed the first diagnostic criteria for Asians. These criteria were revised in 2019 and are currently used in Asia [3]. Specifically, grip strength measurements assess muscle strength, 6 m walk speed evaluates physical performance, and dual-energy X-ray absorptiometry (DXA) and bioelectrical impedance analysis (BIA) are used to measure skeletal muscle mass, investigating the presence of skeletal muscle loss. A diagnosis of sarcopenia is made when low muscle mass is combined with either low muscle strength or low physical performance. Instead of the 6 m walk test, the five-time chair stand test or the Short Physical Performance Battery (SPPB) score, which includes three tasks (walking, sit-to-stand, and standing balance), may be used.

Chronic kidney disease (CKD) was defined by the Kidney Disease Outcomes Quality Initiative (KDOQI) in 2002. As CKD progresses, it often leads to end-stage kidney disease, requiring treatments like dialysis or kidney transplantation. Furthermore, it increases the risk of cardiovascular diseases, including myocardial infarction (MI) and stroke, in addition to culminating in mortality. The prevalence of CKD is growing not just in Japan but worldwide, making prevention crucial. Lifestyle improvements are key in managing CKD. Nutritional and dietary counseling, especially about protein and sodium intake, are vital, and dietitians play a pivotal role in slowing kidney function decline.

Several studies have reported that CKD is associated with sarcopenia. According to the National Health Interview Survey (NHIS), the appendicular lean mass index (ALMI) in patients with stage G4 CKD is 2.58-fold lower than that in non-CKD patients, as measured by DXA [4]. Among Japanese patients with conservative CKD, 57.3% exhibit a diminished ALMI, with 25% concurrently suffering from sarcopenia [5]. Furthermore, the presence of sarcopenia is closely linked to increased mortality rates in CKD patients; therefore, it is important to explore the relationship between sarcopenia and CKD [6]. The precise mechanisms underlying the association between renal dysfunction and muscle weakness remain unclear. However, it has been postulated that factors such as stringent dietary constraints, uremic toxins, inflammation, endocrine disorders, and metabolic acidosis collectively influence protein synthesis, protein degradation, and nerve–muscle integrity, culminating in the loss of muscle mass and strength [7,8]. In particular, several reports indicate that protein-restricted diets can delay the progression of renal function deterioration, but there are also reports that excessive protein restriction contributes to sarcopenia in elderly patients with CKD [9,10,11].

Certain nutrients and dietary patterns have been shown to prevent age-related decline in muscle strength and function. Several studies have identified correlations between the consumption of milk-based products, adherence to the Mediterranean Diet, and adoption of plant-based diets with reduced rates of muscle strength decline and impaired physical function [12,13,14,15]. For example, Kim et al. studied the relationship between vegetable and fruit consumption and the occurrence of sarcopenia in older individuals. They used cross-sectional data and focused on a community-dwelling cohort of men and women aged 65 and older. The findings revealed that, in men, consuming vegetables, fruits, or both was significantly associated with a reduced risk of sarcopenia. For women, higher fruit consumption correlated with a lower risk of sarcopenia [13]. However, to the best of our knowledge, no studies have investigated the relationship between muscle strength and dietary patterns among CKD patients.

Therefore, in this cross-sectional study, we aimed to investigate the relationship between sarcopenia and dietary patterns among patients with CKD, particularly elderly patients.

## 2. Materials and Methods

### 2.1. Study Participants

This study involved a post hoc analysis of data derived from a previously performed cross-sectional study: the JUSTICE-TOKYO (JUntendo Sarcopenia Region for Exploring Predictors and Prognosis in the Elderly in TOKYO) study (*n* = 1078) (UMIN0000 43133). The JUSTICE-TOKYO study was a single-center prospective observational study that included elderly patients selected from a large cohort of outpatients at our center. It was conducted to explore the clinical significance of frailty and sarcopenia. The JUSTICE-TOKYO study adhered to the principles of the Declaration of Helsinki and was approved by the hospital ethics committee. All participants provided informed consent forms and signed them prior to enrollment in the study. The registration process of the study involved recording the patients’ profiles, medical histories, blood test results, physical and skeletal muscle mass measurements, physiological function test results, and nutritional assessment results. Data were entered prospectively into Research Electronic Data Capture (REDCap, version 12.5.9), a web-based software solution that allows researchers to create secure online forms.

Of all participants in the JUSTICE-TOKYO study, 459 with conservative CKD were enrolled in this study. Patients requiring renal replacement therapy were excluded. CKD was defined as an estimated glomerular filtration rate (eGFR) of <60 mL/min/1.73 m^2^ present for >3 months. CKD stages were classified based on the eGFR according to the Kidney Disease Improving Global Outcomes (KDIGO) guidelines, as follows: stage 3 (CKD G3), 30–59 mL/min/1.73 m^2^; stage 4 (CKD G4), 15–29 mL/min/1.73 m^2^; and stage 5 (CKD G5), <15 mL/min/1.73 m^2^. The eGFR was calculated using the Chronic Kidney Disease Epidemiology Collaboration (CKD-EPI) calculator without removing the body surface adjustment. Owing to missing data, 18 patients were excluded, and data from the remaining 441 patients were used for the analysis (Figure S1).

### 2.2. General Assessment

Body mass index (BMI) was calculated by dividing body weight by the square of body height, expressed in meters (kg/m^2^). The Brinkman index score was determined by multiplying the number of cigarettes smoked per day by the number of years the individual has smoked. Drinking frequency was categorized as rarely, 1–4 days per week, or 5–7 days per week. Alcohol consumption was assigned values: 0 for rarely, 1 for 1–4 days per week, and 2 for 5–7 days per week. The average alcohol consumption was then calculated. Information regarding ongoing/history of disease, Brinkman index, and alcohol consumption was obtained through interviews conducted by the chief physician of outpatient services across various internal medicine departments (gastroenterology, cardiology, respiratory medicine, and rheumatology). Blood pressure measurements were manually performed by a nurse at our center, either on the left or right upper arm.

### 2.3. Laboratory Parameters

Morning blood samples were collected after an overnight fast. The samples were then divided and injected into blood collection tubes, which were transported to the Department of Clinical Laboratory at our center within 20 min. Serum creatinine, serum sodium, serum potassium, serum chloride, serum albumin, and glycated hemoglobin (HbA1c) levels were analyzed using certified methods.

### 2.4. Definition of Sarcopenia

Sarcopenia was defined using the diagnostic algorithm recommended by the AWGS 2019 Consensus Update on Sarcopenia Diagnosis and Treatment [3], which assesses the presence of low muscle function (low muscle strength or low physical performance) and low muscle mass, as mentioned above.

In the present study, subjects ≥ 65 years old were considered to have sarcopenia if they exhibited low appendicular skeletal muscle mass with either low handgrip strength or slow gait speed. Handgrip strength, gait speed, and muscle mass were measured. Handgrip strength was measured using a handgrip dynamometer (Toei Light Co. Ltd., Saitama, Japan). Both hands were tested twice with a 30 s rest between measurements, and the highest value was recorded as maximum muscle strength. Low grip strength was determined according to the sex-specific cutoff for maximum muscle strength according to the AWGS criteria (<18 kg in women and <28 kg in men). To assess physical performance through gait speed, a 6 m walk test was administered. Participants were instructed to walk as quickly as possible along a flat, straight path within the hospital. The time required to cover the 6 m was manually recorded using a stopwatch. Slow gait speed was defined as gait speed < 1.0 m/s according to the AWGS criteria. Regional fat mass and lean mass were measured using whole-body DXA (Prodigy Advance, GE Healthcare, Madison, WI, USA). Participants were positioned for whole-body scans in accordance with the manufacturer’s protocol. Whole-body fat mass and lean mass were divided into several regions, including the arms, legs, and trunk. Appendicular lean mass was estimated as the sum of the lean mass of the upper and lower limbs. The appendicular skeletal muscle mass index (SMI) was calculated as appendicular lean mass divided by the square of height (kg/m^2^). Low appendicular skeletal muscle mass was defined as SMI < 5.4 kg/m^2^ in women and <7.0 kg/m^2^ in men.

### 2.5. Nutritional Evaluation

Various methods are used to evaluate dietary patterns. However, as food culture differs from country to country, it is necessary to use an evaluation method that reflects the dietary habits of the Japanese. Therefore, dietary patterns were assessed using the dietary variety score (DVS) [16]. The DVS is a commonly employed metric for assessing the diversity of food intake in Japan’s elderly population and has been observed to be associated with physical function, body composition, risk of falls, and sarcopenia in older adults [17,18,19,20]. A survey was administered to determine the frequency of the consumption of 10 food groups, including fish/shellfish, meat, eggs, milk, soy products, green/yellow vegetables (GYV), seaweed, fruits, potatoes, and fats/oils. A score of 1 was assigned for “almost every day”, while a score of 0 was given for “once every 2 days”, “once or twice a week”, or “almost never”. The DVS was calculated as the sum of the scores for all 10 food items, with a maximum achievable score of 10. Participants provided their own responses to the questionnaire.

### 2.6. Statistical Analysis

Age, BMI, systolic blood pressure (SBP), diastolic blood pressure (DBP), eGFR, serum sodium, serum potassium, serum chloride, serum albumin, HbA1c, SMI, grip strength, gait speed, and DVS were reported as mean ± standard deviation. Female sex, the proportion of patients with CKD G3, CKD G4-5, smoking, drinking, history of MI, and history of stroke were presented as percentages. Smoking was defined as a Brinkman index ≥ 400. Drinking was defined as consuming alcohol one or more times per week. The chi-square test was used for categorical variables, and the two-sample t-test was used for continuous variables. Additionally, multivariate cross-sectional logistic regression analysis was conducted to assess the associations between different variables and sarcopenia, with odds ratios (ORs) and 95% confidence intervals (CIs) estimated. Multicollinearity was examined for robustness begore the regression analysis. K-means cluster analysis, an unsupervised machine learning algorithm, was applied to group patients from both groups into clusters based on their DVS. All statistical analyses were performed using SPSS version 26 software (IBM Corporation, Armonk, NY, USA). Statistical significance was set at *p* < 0.05.

## 3. Results

Table 1 presents the characteristics of the study participants in both the overall group and the sarcopenia and non-sarcopenia subgroups. The average age of the overall population was 79.7 ± 5.9 years, and 54.9% of the participants were women. Moreover, 93.2% of the participants had CKD G3. The mean concentrations of serum sodium, potassium, chloride, and albumin were all within reference values. Among all participants, 100 (22.7%) were diagnosed with sarcopenia. In the sarcopenia group, SMI was 5.02 ± 0.34 kg/m^2^ in women and 6.17 ± 0.63 kg/m^2^ in men. Grip strength was 16.4 ± 3.1 kg in women and 26.7 ± 5.9 kg in men, while gait speed was 0.9 ± 0.3 m/s. When comparing the sarcopenia and non-sarcopenia subgroups, the sarcopenia group was characterized by significantly older age and larger proportion of smokers and patients with a history of stroke. Conversely, the non-sarcopenia group had notably larger proportions of women and patients with CKD G3, and the BMI, DBP, and serum albumin levels in this group were higher than those in the sarcopenia group. Notably, no significant differences in DVS were observed between the two groups.

Table 2 presents the results of the univariate analysis of the consumption of each food item, such as fish, meat, eggs, and others, between the sarcopenia and non-sarcopenia groups. The proportion of patients who reported consuming GYV “almost daily” (score: 1) was significantly larger in the non-sarcopenia group (54.0% vs. 70.4%, *p* = 0.002). There were no significant differences in the consumption of other food items between the groups. Subsequent logistic regression analysis with sarcopenia as the dependent variable and age, proportion of women, BMI, DBP, eGFR, serum albumin level, HbA1c, smoking status, stroke, and DVS for each food group as independent variables was performed. The results of the analysis are presented in Table 3 and Figure S2. Advanced age (OR: 1.093, 95% CI: 1.036–1.152) and increased HbA1c levels (OR: 1.728, 95% CI: 1.160–2.574) were associated with sarcopenia. Among dietary patterns, only consuming GYV almost daily was associated with non-sarcopenia (OR: 0.414, 95% CI: 0.212–0.806).

Next, since the population included in this study consisted mostly of CKD G3 patients (93.2%), the analysis was divided into CKD G3 and CKD G4-5 patients by stage, taking into account the impact of CKD G4-5 patients on the overall results. In terms of patient background, the sarcopenia group had a significantly lower proportion of women, BMI, and serum albumin levels among patients with CKD G3 (Table 4). No significant differences in DVS were found between the sarcopenia and non-sarcopenia groups among those patients. When the 10 foods based on DVS were analyzed by CKD stage (Table 5), a significantly higher percentage of CKD G3 patients in the non-sarcopenia group consumed GYV almost daily (54.7% vs. 70.8%, *p* = 0.005), while no significant difference was found between the groups in CKD G4-5 patients. Therefore, a multivariate analysis was performed on CKD G3 patients only, and as shown in Table 6, frequent intake of GYV was statistically significantly associated with non-sarcopenia (OR: 0.350, 95% CI: 0.176–0.695), similar to the results for CKD overall.

To assess the dietary patterns of individuals with and without sarcopenia, k-means cluster analysis was conducted in both groups among participants with CKD G3 only. After careful deliberation, a reasonably large three-cluster solution (>10% of the sample size) was chosen. Table 7 presents the results of the analysis. The results in both groups indicated that the cluster characterized by a lower DVS was designated as “low”, the one with a moderate DVS was labeled as “moderate”, and the cluster with a higher DVS was denoted as “high” (Table 7). In the sarcopenia group, the “low” cluster was most prevalent, followed by “moderate” and “high” (*n* = 32, *n* = 27, and *n* = 26, respectively). Among patients in the non-sarcopenia group, the most common cluster was “low”, followed by “moderate” and “high” (*n* = 165, *n* = 92, and *n* = 68, respectively; Table 7). When comparing the “low” cluster between the two groups in terms of DVS and consumption of each food item, the non-sarcopenia group had a significantly larger proportion of patients who consumed milk (25.0% vs. 44.8%, *p* = 0.037), fruits (0.0% vs. 40.0%, *p* < 0.001), and GYV (21.9% vs. 52.7%, *p* = 0.001) almost daily. Additionally, the DVS in the non-sarcopenia group was higher than that in the sarcopenia group (1.28 ± 1.30 vs. 2.26 ± 1.28, *p* < 0.001). Conversely, the sarcopenia group had a significantly larger proportion of individuals who consumed eggs (15.6% vs. 2.4%, *p* = 0.001) almost daily.

## 4. Discussion

In the present study, we examined 441 patients diagnosed with CKD (mean age: 79.7 ± 5.9 years, eGFR: 48.1 ± 11.0 mL/min/1.73 m^2^) to investigate the association between sarcopenia and dietary patterns. Initially, we divided the participants into sarcopenia and non-sarcopenia groups. Our analysis aimed to identify disparities in DVS and dietary patterns between the two groups. We found no significant differences in the DVS between these groups; however, a notably higher frequency of GYV consumption was observed in the non-sarcopenia group.

Subsequently, a k-means cluster analysis was conducted, classifying patients in both groups into three distinct clusters based on their DVS: “low”, “moderate”, and “high”. The “low” cluster, which had the lowest DVS, included the largest proportion of patients from both groups. The proportion of patients who reported consuming GYV, as well as eggs, milk, and fruits almost daily, was significantly lower in the “low” cluster of the sarcopenia group than in the non-sarcopenia group. Our results suggest that consuming a varied diet, particularly one rich in GYV, is associated with a lower risk of sarcopenia.

Protein restriction has been considered renoprotective and is a standard treatment for CKD [21,22]. However, excessive protein restriction has been linked to muscle weakness, leading to discussions about relaxing such restrictions in CKD patients with sarcopenia [23]. Thus, treating these patients presents a dilemma: protein restriction decreases the risk of worsening renal function but increases the risk of sarcopenia.

It is commonly believed that plant-based diets are deficient in essential amino acids, but a study has shown that this is not necessarily the case [24]. For example, the European Prospective Investigation into Cancer and Nutrition (EPIC) in Oxford and the California Seventh-Day Adventist cohorts have shown that a balanced and diverse plant-based diet is nutritionally adequate and beneficial [25,26]. Patients with CKD G3-4 who consumed a vegan diet consisting of a combination of cereals and legumes, pre-constituted to contain all essential amino acids, showed no signs of nutritional deficiency 13 months post-assessment [27], and it has been suggested that a Mediterranean Diet, high in vegetables, legumes, and cereals, may help slow CKD progression and prevent complications [28]. In our study, multivariate analysis revealed a higher frequency of GYV and soy product intake in the non-sarcopenia group. However, no differences in fish, meat, egg, or milk intake were observed between the two groups. These findings do not fully support a vegan diet but do suggest that a well-balanced plant-based diet might mitigate muscle weakness and decrease the risk of renal function decline and sarcopenia, while adhering to protein restrictions.

The protective effects of vegetable-based diets on muscle strength are not fully understood; however, one theory posits that alkaline diets, such as those rich in vegetables, counteract metabolic acidosis linked to muscle wasting, thereby preserving muscle mass. Some studies have provided evidence supporting this hypothesis, such as a randomized controlled trial of 162 healthy individuals over 50 years old showing that sodium bicarbonate supplementation improved leg press power in women [29]. A longitudinal cohort study of 384 individuals aged 65 years and older found that a diet high in potassium and low in dietary acid load could preserve muscle mass [30]. Additionally, a cross-sectional study of 2176 Japanese women aged 65–94 years indicated that a higher dietary acid load was positively associated with frailty [31]. The International Olympic Committee recognizes sodium bicarbonate as a supplement with substantial evidence for enhancing performance in certain scenarios [32]. While the precise mechanisms by which vegetable-based diets affect muscle mass and counter sarcopenia and frailty require further research, existing evidence highlights their potential importance. Randomized controlled trials are needed to validate these relationships and elucidate their underlying mechanisms.

This study has several limitations that should be considered. First, the cross-sectional design has inherent limitations in establishing causal relationships. Therefore, prospective investigations are imperative for validating the causal link between sarcopenia and dietary factors in the elderly population. Second, most participants (>90%) had CKD G3, with only a small subset (6.8%) having CKD G4-5. Given the need to manage potassium-rich foods and the risk of hyperkalemia in patients with CKD, our study did not find evidence to suggest that a plant-based diet, particularly one involving vegetables and fruits, significantly increased the risk of hyperkalemia. The present study did not investigate whether the presence of comorbidities or other medical conditions among participants influenced dietary choices. It cannot be ruled out that these factors may have had an impact on dietary patterns. Consequently, our findings may not be generalizable to all patients with CKD. Lastly, our study focused only on dietary patterns and did not evaluate the daily nutritional intake of the participants; thus, total calorie and protein intake cannot be determined solely from the DVS. To our knowledge, no other report has explored the relationship between dietary patterns and sarcopenia in elderly patients with conservative CKD. Dietary patterns were assessed through a questionnaire, which healthcare professionals or dietitians can easily and objectively evaluate. The findings suggest that dietary diversity could be a key factor in the nutritional assessment and dietary support for elderly CKD patients with sarcopenia. This warrants further improvement in follow-up study designs.

## 5. Conclusions

In conclusion, this study examined the association between dietary patterns and sarcopenia in 441 patients with CKD. The results suggest the potential of frequent GYV consumption to mitigate the risk of sarcopenia. Future prospective and interventional studies are warranted to analyze the causal relationship between vegetable-based diets and the risk of sarcopenia.

## Figures and Tables

**Table 1:** Characteristics of participants stratified by sarcopenia status (*n* = 441).

		Overall (*n* = 441)	Sarcopenia (*n* = 100)	Non-Sarcopenia (*n* = 341)	*p*-Value *
Age, mean ± SD, years		79.7 ± 5.9	81.6 ± 5.7	79.2 ± 5.8	<0.001
Female, *n* (%)		242 (54.9)	34 (34.0)	208 (61)	<0.001
CKD G3, *n* (%)		411 (93.2)	86 (86.0)	325 (95.3)	0.001
CKD G4-5, *n* (%)		30 (6.8)	14 (14.0)	16 (4.7)	0.001
BMI, mean ± SD, kg/m^2^		23.2 ± 3.7	20.8 ± 3.6	23.9 ± 3.4	<0.001
SBP, mean ± SD, mmHg		137.9 ± 19.3	136.0 ± 18.8	138.5 ± 19.5	0.246
DBP, mean ± SD, mmHg		77.4 ± 12.2	75.0 ± 12.7	78.0 ± 12.0	0.027
eGFR, mean ± SD, mL/min/1.73m^2^		48.1 ± 11.0	46.5 ± 13.1	48.6 ± 10.3	0.145
Na, mean ± SD, mmol/L		141.4 ± 2.5	141.1 ± 2.8	141.5 ± 2.4	0.125
K, mean ± SD, mmol/L		4.3 ± 0.4	4.3 ± 0.5	4.3 ± 0.4	0.983
Cl, mean ± SD, mmol/L		104.3 ± 3.0	104.1 ± 3.8	104.3 ± 2.7	0.559
Albumin, mean ± SD, g/dL		4.1 ± 0.4	4.0 ± 0.4	4.1 ± 0.3	0.034
HbA1c, mean ± SD, %		6.1 ± 0.8	6.2 ± 0.9	6.1 ± 0.8	0.326
Smoking (BI > 400), *n* (%)		147 (33.3)	45 (45.0)	102 (29.9)	0.005
Drinking, *n* (%)		124 (28.1)	28 (28.0)	96 (28.2)	0.976
MI, *n* (%)		23 (5.2)	6 (6.0)	17 (5.0)	0.688
Stroke, *n* (%)		36 (8.2)	13 (13.0)	23 (6.7)	0.044
SMI, mean ± SD, kg/m^2^	female	5.91 ± 0.71	5.02 ± 0.34	6.05 ± 0.65	<0.001
	male	6.86 ± 0.82	6.17 ± 0.63	7.20 ± 0.67	<0.001
Grip Strength, mean ± SD, kg	female	20.1 ± 4.7	16.4 ± 3.1	20.7 ± 4.7	<0.001
	male	32.1 ± 7.1	26.7 ± 5.9	34.8 ± 6.0	<0.001
Walking Speed, mean ± SD, m/s		1.2 ± 0.4	0.9 ± 0.3	1.2 ± 0.4	<0.001
DVS, mean ± SD		3.7 ± 2.2	3.4 ± 2.4	3.8 ± 2.2	0.121

* Comparisons with sarcopenia and non-sarcopenia groups; *p* < 0.05 considered significant. CKD, chronic kidney disease; SD, standard deviation; BMI, body mass index; SBP, systolic blood pressure; DBP, diastolic blood pressure; BI, Brinkman index; MI, myocardial infarction; SMI, skeletal muscle mass index; DVS, Dietary Variety Score.

**Table 2:** Daily consumption proportions across 10 food groups based on DVS.

	Sarcopenia (*n* = 100)	Non-Sarcopenia (*n* = 341)	*p*-Value
Fish intake: almost daily, *n* (%)	31 (31.0)	110 (32.3)	0.813
Meat intake: almost daily, *n* (%)	20 (20.0)	83 (24.3)	0.367
Eggs intake: almost daily, *n* (%)	37 (37.0)	129 (37.8)	0.88
Milk intake: almost daily, *n* (%)	42 (42.0)	174 (51.0)	0.112
Soy products intake: almost daily, *n* (%)	34 (34.0)	150 (44.0)	0.075
GYV intake: almost daily, *n* (%)	54 (54.0)	240 (70.4)	0.002
Seaweed intake: almost daily, *n* (%)	21 (21.0)	76 (22.3)	0.785
Potatoes intake: almost daily, *n* (%)	12 (12.0)	35 (10.3)	0.621
Fruits intake: almost daily, *n* (%)	53 (53.0)	182 (53.4)	0.948
Fats/Oils intake: almost daily, *n* (%)	40 (40.0)	128 (37.5)	0.656

*p* < 0.05 considered significant. DVS, Dietary Variety Score; GYV, green/yellow vegetables.

**Table 3:** Logistic regression analysis for factors associated with sarcopenia.

Variables	OR	95% CI	*p*-Value
Age	1.093	1.036–1.152	0.001
Female	0.210	0.103–0.428	<0.001
BMI	0.652	0.584–0.728	<0.001
DBP	0.990	0.966–1.014	0.401
eGFR	0.988	0.964–1.013	0.345
Albumin	0.777	0.364–1.658	0.513
HbA1c	1.728	1.160–2.574	0.007
Smoking	1.136	0.570–2.264	0.717
Stroke	1.650	0.646–4.217	0.295
Fish intake: almost daily	0.734	0.366–1.473	0.384
Meat intake: almost daily	0.763	0.365–1.598	0.474
Eggs intake: almost daily	1.319	0.709–2.455	0.381
Milk intake: almost daily	0.611	0.342–1.093	0.097
Soy products intake: almost daily	0.549	0.296–1.019	0.057
GYV intake: almost daily	0.414	0.212–0.806	0.009
Seaweed intake: almost daily	1.604	0.733–3.511	0.237
Potatoes intake: almost daily	0.921	0.344–2.467	0.871
Fruits intake: almost daily	1.362	0.752–2.464	0.308
Fats/Oils intake: almost daily	1.203	0.662–2.187	0.544

*p* < 0.05 considerd significant. OR, odds ratio; CI, confidence interval; BMI, body mass index; DBP, diastolic blood pressure; GYV, green/yellow vegetables.

**Table 4:** Characteristics of participants stratified by CKD stages and sarcopenia status.

		CKD G3 (*n* = 411)			CKD G4-5 (*n* = 30)		
		Sarcopenia (*n* = 86)	Non-Sarcopenia (*n* = 325)	*p*-Value	Sarcopenia (*n* = 14)	Non-Sarcopenia (*n* = 16)	*p*-Value
Age, mean ± SD, years		81.7 ± 5.3	79.1 ± 5.8	<0.001	80.6 ± 7.5	80.1 ± 5.9	0.837
Female, *n* (%)		28 (32.6)	198 (60.9)	<0.001	6 (42.9)	10 (62.5)	0.282
BMI, mean ± SD, kg/m^2^		20.9 ± 3.0	23.8 ± 3.4	<0.001	20.3 ± 6.2	25.4 ± 3.7	0.010
SBP, mean ± SD, mmHg		135.6 ± 18.3	138.6 ± 19.1	0.188	138.1 ± 22.6	136.1 ± 26.5	0.825
DBP, mean ± SD, mmHg		75.5 ± 13.1	78.1 ± 12.1	0.082	71.5 ± 8.7	76.1 ± 10.3	0.198
eGFR, mean ± SD, mL/min/1.73 m^2^		50.9 ± 7.0	50.1 ± 7.6	0.428	19.7 ± 9.1	17.0 ± 7.5	0.380
Na, mean ± SD, mmol/L		141.2 ± 2.6	141.6 ± 2.3	0.244	140.1 ± 3.4	140.1 ± 2.5	0.986
K, mean ± SD, mmol/L		4.3 ± 0.4	4.3 ± 0.4	0.592	4.6 ± 0.8	4.6 ± 0.4	0.744
Cl, mean ± SD, mmol/L		104.1 ± 3.2	104.3 ± 2.6	0.502	103.8 ± 6.4	103.7 ± 4.0	0.958
Albumin, mean ± SD, g/dL		4.0 ± 0.4	4.1 ± 0.3	0.044	4.0 ± 0.4	3.8 ± 0.5	0.238
HbA1c, mean ± SD, %		6.2 ± 0.9	6.1 ± 0.8	0.291	6.2 ± 0.9	6.3 ± 1.0	0.673
Smoking (BI > 400), *n* (%)		39 (45.3)	95 (29.2)	0.005	6 (42.9)	7 (43.8)	0.961
Drinking, *n* (%)		24 (27.9)	92 (28.3)	0.941	4 (28.6)	4 (25.0)	0.825
MI, *n* (%)		3 (3.5)	15 (4.6)	0.650	3 (21.4)	2 (12.5)	0.513
Stroke, *n* (%)		9 (10.5)	21 (6.5)	0.204	4 (28.6)	2 (12.5)	0.272
SMI, mean ± SD, kg/m^2^	female	4.97 ± 0.33	6.04 ± 0.65	<0.001	5.26 ± 0.26	6.32 ± 0.53	<0.001
	male	6.16 ± 0.62	7.21 ± 0.68	<0.001	6.28 ± 0.70	7.01 ± 0.44	0.044
Grip Strength, mean ± SD, kg	female	16.2 ± 2.8	20.9 ± 4.7	<0.001	17.3 ± 4.7	17.3 ± 2.6	0.986
	male	26.5 ± 6.0	35.0 ± 5.9	<0.001	28.4 ± 5.4	31.2 ± 8.3	0.461
Walking Speed, mean ± SD, m/s		1.0 ± 0.3	1.2 ± 0.4	<0.001	0.9 ± 0.3	1.0 ± 0.3	0.458
DVS, mean ± SD		3.4 ± 2.4	3.8 ± 2.2	0.115	3.6 ± 2.6	3.8 ± 1.8	0.837

*p* < 0.05 considered significant. CKD, chronic kidney disease; SD, standard deviation; BMI, body mass index; SBP, systolic blood pressure; DBP, diastolic blood pressure; BI, Brinkman index; MI, myocardial infarction; SMI, skeletal muscle mass index; DVS, Dietary Variety Score.

**Table 5:** Daily consumption proportions across 10 food groups based on DVS among patients with CKD G3 and G4-5.

	CKD G3 (*n* = 411)			CKD G4-5 (*n* = 30)		
	Sarcopenia (*n* = 86)	Non-Sarcopenia (*n* = 325)	*p*-Value	Sarcopenia (*n* = 14)	Non-Sarcopenia (*n* = 16)	*p*-Value
Fish intake: almost daily, *n* (%)	27 (31.4)	104 (32.0)	0.915	4 (28.6)	6 (37.5)	0.605
Meat intake: almost daily, *n* (%)	15 (17.4)	78 (24.0)	0.196	5 (35.7)	5 (31.3)	0.796
Eggs intake: almost daily, *n* (%)	33 (38.4)	119 (36.6)	0.764	4 (28.6)	10 (62.5)	0.630
Milk intake: almost daily, *n* (%)	36 (41.9)	168 (51.7)	0.105	6 (42.9)	6 (37.5)	0.765
Soy products intake: almost daily, *n* (%)	29 (33.7)	145 (44.6)	0.069	5 (35.7)	5 (31.3)	0.796
GYV intake: almost daily, *n* (%)	47 (54.7)	230 (70.8)	0.005	7 (50.0)	10 (62.4)	0.491
Seaweed intake: almost daily, *n* (%)	18 (21.0)	72 (22.1)	0.807	3 (21.4)	4 (25.0)	0.818
Potatoes intake: almost daily, *n* (%)	10 (11.6)	33 (10.2)	0.691	2 (14.3)	2 (12.5)	0.886
Fruits intake: almost daily, *n* (%)	46 (53.5)	175 (53.8)	0.953	7 (50.0)	7 (43.8)	0.732
Fats/Oils intake: almost daily, *n* (%)	32 (37.2)	122 (37.5)	0.985	8 (57.1)	6 (37.5)	0.282

*p* < 0.05 considered significant. CKD, chronic kidney disease; DVS, Dietary Variety Score; GYV, green/yellow vegetables.

**Table 6:** Logistic regression analysis for factors associated with sarcopenia among participants with CKD G3.

Variables	OR	95% CI	*p*-Value
Age	1.103	1.042–1.167	0.001
Female	0.166	0.086–0.320	<0.001
BMI	0.663	0.590–0.745	<0.001
Albumin	0.628	0.255–1.543	0.310
Smoking	2.201	1.196–4.050	0.011
Fish intake: almost daily	1.176	0.572–2.420	0.659
Meat intake: almost daily	0.548	0.244–1.233	0.146
Eggs intake: almost daily	1.198	0.618–2.323	0.594
Milk intake: almost daily	0.764	0.419–1.390	0.594
Soy products intake: almost daily	0.623	0.327–1.187	0.150
GYV intake: almost daily	0.350	0.176–0.695	0.003
Seaweed intake: almost daily	1.414	0.642–3.114	0.389
Potatoes intake: almost daily	1.604	0.580–4.437	0.363
Fruits intake: almost daily	1.417	0.744–2.698	0.289
Fats/Oils intake: almost daily	1.294	0.690–2.427	0.421

*p* < 0.05 considered significant. OR, odds ratio; CI, confidence interval; BMI, body mass index; DBP, diastolic blood pressure; GYV, green/yellow vegetables.

**Table 7:** Profile of three dietary patterns identified by cluster analysis in sarcopenia and non-sarcopenia group among the patients with CKD G3.

	Sarcopenia (*n* = 85)			Non-Sarcopenia (*n* = 341)					
	Low (*n* = 32)	Moderate (*n* = 27)	High (*n* = 26)	Low (*n* = 165)	Moderate (*n* = 92)	High (*n* = 68)	*p*-Value ^a^	*p*-Value ^b^	*p*-Value ^c^
Fish intake: almost daily, *n* (%)	3 (9.0)	10 (37.0)	13 (50.0)	19 (11.5)	30 (32.6)	55 (80.9)	0.725	0.668	0.003
Meat intake: almost daily, *n* (%)	1 (3.1)	2(7.4)	11 (42.3)	16 (9.7)	13 (14.1)	49 (72.1)	0.226	0.355	0.007
Eggs intake: almost daily, *n* (%)	5 (15.6)	18 (54.5)	23 (88.5)	4 (2.4)	92 (100.0)	23 (33.8)	0.001	<0.001	<0.001
Milk intake: almost daily, *n* (%)	8 (25.0)	10 (37.0)	18 (69.2)	74 (44.8)	47 (55.3)	42 (61.8)	0.037	0.199	0.500
Soy products intake: almost daily, *n* (%)	5 (15.6)	6 (22.2)	17 (65.4)	46 (27.9)	52 (56.5)	50 (73.5)	0.148	0.002	0.435
GYV intake: almost daily, *n* (%)	7 (21.9)	14 (51.9)	25 (96.2)	87 (52.7)	49 (53.3)	68 (100.0)	0.001	0.897	0.104
Seaweed intake: almost daily, *n* (%)	1 (3.1)	2 (7.4)	15 (57.7)	7 (4.2)	75 (81.5)	42 (61.8)	0.770	<0.001	0.718
Potatoes intake: almost daily, *n* (%)	2 (6.3)	0 (0.0)	7 (26.9)	6 (3.6)	23 (25.0)	17 (25.0)	0.493	0.004	0.848
Fruits intake: almost daily, *n* (%)	0 (0.0)	27 (100.0)	19 (73.1)	66 (40.0)	10 (10.9)	54 (79.4)	<0.001	<0.001	0.510
Fats/Oils intake: almost daily, *n* (%)	9 (28.1)	8 (27.0)	15 (57.7)	48 (29.1)	55 (59.8)	40 (58.8)	0.912	0.006	0.921
DVS, mean ± SD	1.28 ± 1.30	3.11 ± 1.05	6.27 ± 1.34	2.26 ± 1.28	4.70 ± 1.38	6.47 ± 1.50	<0.001	<0.001	0.551

^a^ Compared with “Low” clusters, ^b^ compared with “Moderate” clusters, ^c^ compared with “High” clusters in sarcopenia and non-sarcopenia group, *p* < 0.05 was significant; GYV, green/yellow vegetables; DVS, Dietary Variety Score; SD, standard deviation.

## Data Availability

The datasets generated and/or analyzed during the current study are available within the article and its Supplementary Materials.

## Supplementary Materials

The following supporting information can be downloaded at: https://www.mdpi.com/article/10.3390/nu15234994/s1, Figure S1: Results of the logistic regression analysis related to factors associated with sarcopenia; Figure S2: Profile of three dietary patterns identified by cluster analysis in both sarcopenia and non-sarcopenia groups.
