# Supplementary material for: Cross-Sectional Study on the Association between Dietary Patterns and Sarcopenia in Elderly Patients with Chronic Kidney Disease Receiving Conservative Treatment
Source: Nutrients. 2023 Dec 1;15(23):4994. doi: 10.3390/nu15234994 (PMC10708199; doi:10.3390/nu15234994)
Supplement: Supplementary file 1 [file nutrients-15-04994-s001.zip › nutrients-2702147-supplementary.pdf]

nutrients

Supplemental Materials

Cross-Sectional Study on the Association between Dietary Patterns and Sarcopenia in Elderly Patients with Chronic Kidney Disease Receiving Conservative Treatment

Hiroyuki Inoshita [1,*], Daisuke Asaoka [2], Kei Matsuno [3], Naotake Yanagisawa [4], Yusuke Suzuki [5] and Katsumi Miyauchi [6]

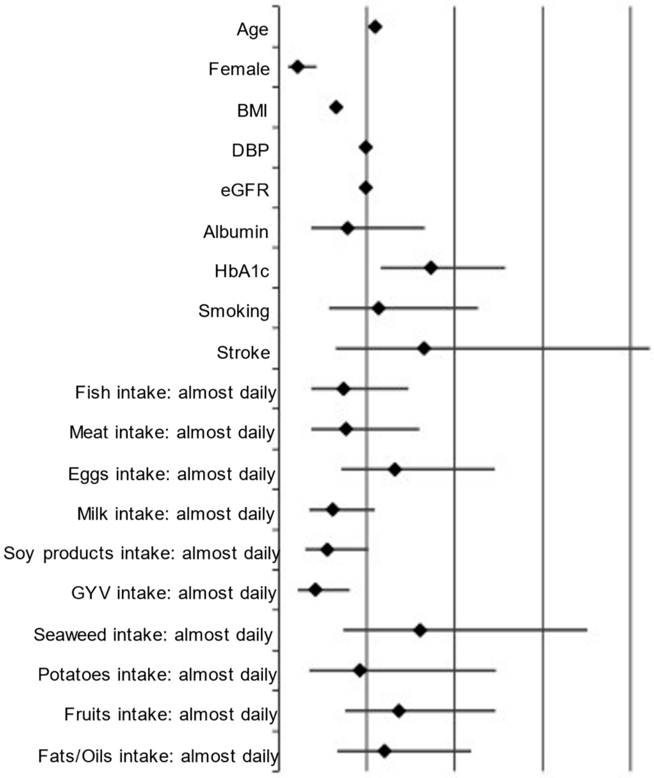

	OR	95% CI	p value
Age	1.093	1.036 - 1.152	0.001
Female	0.210	0.103 - 0.428	<0.001
BMI	0.652	0.584 - 0.728	<0.001
DBP	0.990	0.966 - 1.014	0.401
eGFR	0.988	0.964 - 1.013	0.345
Albumin	0.777	0.364 - 1.658	0.513
HbA1c	1.728	1.160 - 2.574	0.007
Smoking	1.136	0.570 - 2.264	0.717
Stroke	1.650	0.646 - 4.217	0.295
Fish intake: almost daily	0.734	0.366 - 1.473	0.384
Meat intake: almost daily	0.763	0.365 - 1.598	0.474
Eggs intake: almost daily	1.319	0.709 - 2.455	0.381
Milk intake: almost daily	0.611	0.342 - 1.093	0.097
Soy products intake: almost daily	0.549	0.296 - 1.019	0.057
GYV intake: almost daily	0.414	0.212 - 0.806	0.009
Seaweed intake: almost daily	1.604	0.733 - 3.511	0.237
Potatoes intake: almost daily	0.921	0.344 - 2.467	0.871
Fruits intake: almost daily	1.362	0.752 - 2.464	0.308
Fats/Oils intake: almost daily	1.203	0.662 - 2.187	0.544

Figure S1. Results of logistic regression analysis for factors associated with sarcopenia among overall.

The result of the logistic regression analysis with sarcopenia as dependent variable, advanced age and increased HbA1c levels were associated with sarcopenia. The female sex, increased BMI, and consuming GYV almost daily were positively associated with non-sarcopenia.

Abbreviations: OR, odds ratio; CI, confidence interval; BMI, body mass index; DBP, diastolic blood pressure; GYV, green/yellow vegetables

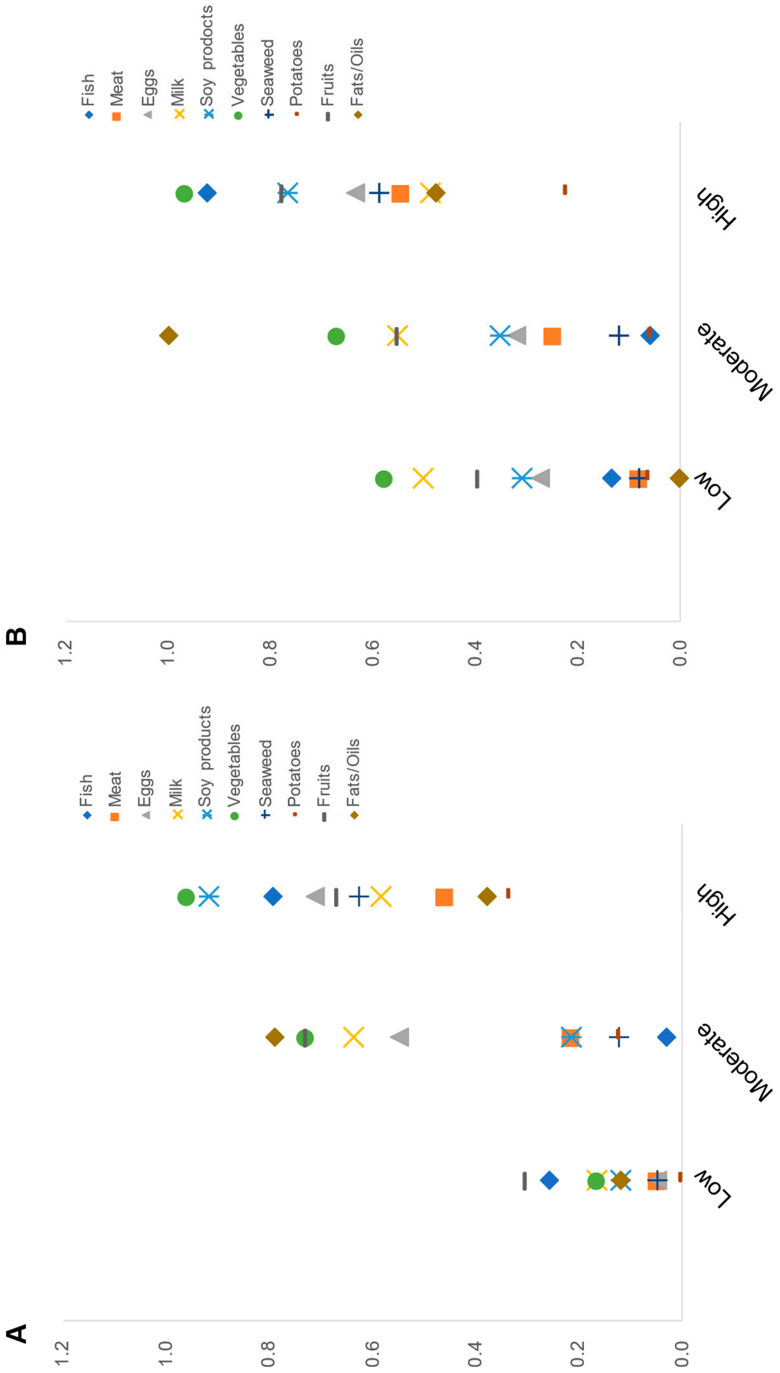

Figure S2. Profile of three dietary patterns identified by cluster analysis in sarcopenia and non-sarcopenia group among overall.

K-means cluster analysis identified three different patterns in (A) sarcopenia and (B) non-sarcopenia group. When comparing the "low" cluster between the two groups in terms of DVS and consumption of each food item, the non-sarcopenia group had a significantly larger proportion of patients who consumed eggs, milk, soy products, and GYV almost daily. Additionally, the DVS in the non-sarcopenia group was higher than that in the sarcopenia group.

Abbreviations: DVS, Dietary Variety Score; GYV, green/yellow vegetables
